# Low rate of severe-end-stage kidney disease after SABR for localised primary kidney cancer

**DOI:** 10.1186/s13014-024-02413-w

**Published:** 2024-02-15

**Authors:** Muhammad Ali, Kendrick Koo, David Chang, Phil Chan, Sheng F. Oon, Daniel Moon, Declan G. Murphy, Renu Eapen, Jeremy Goad, Nathan Lawrentschuk, Arun A. Azad, Sarat Chander, Mark Shaw, Nicholas Hardcastle, Shankar Siva

**Affiliations:** 1 Department of Radiation Oncology, Peter MacCallum Cancer Centre, Melbourne, Australia; 2 Sir Peter MacCallum Department of Oncology, University of Melbourne, Melbourne, Australia; 3 Department of Cancer Imaging, Peter MacCallum Cancer Centre, Melbourne, Australia; 4 Division of Cancer Surgery, Peter MacCallum Cancer Centre, Melbourne, Australia; 5 Department of Surgery, St. Vincent’s Hospital, Melbourne, Australia; 6 Department of Urology, Royal Melbourne Hospital, Melbourne, Australia; 7 Department of Medical Oncology, Peter MacCallum Cancer Centre, Melbourne, Australia; 8 Department of Clinical Pathology, University of Melbourne, Melbourne, Australia; 9 Department of Physical Sciences, Peter MacCallum Cancer Centre, Melbourne, Australia; 10 Centre for Medical Radiation Physics, University of Wollongong, Wollongong, NSW Australia

**Keywords:** Stereotactic ablative radiotherapy, SABR, Renal cell carcinoma, RCC, Chronic kidney disease, End-stage renal disease

## Abstract

**Background:**

Stereotactic ablative body radiotherapy (SABR) is an emerging treatment for patients with primary renal cell carcinoma (RCC). However, its impact on renal function is unclear. This study aimed to evaluate incidence and clinical factors predictive of severe to end-stage chronic kidney disease (CKD) after SABR for RCC.

**Methods and materials:**

This was a Single institutional retrospective analysis of patients with diagnosed primary RCC receiving SABR between 2012–2020. Adult patients with no metastatic disease, baseline estimated glomerular filtration rate (eGFR) of ≥ 30 ml/min/1.73 m^2^, and at least one post-SABR eGFR at six months or later were included in this analysis. Patients with upper tract urothelial carcinoma were excluded. Primary outcome was freedom from severe to end-stage CKD, determined using the Kaplan–Meier estimator. The impact of baseline CKD, age, hypertension, diabetes, tumor size and fractionation schedule were assessed by Cox proportional hazard models.

**Results:**

Seventy-eight consecutive patients were included, with median age of 77.8 years (IQR 70–83), tumor size of 4.5 cm (IQR 3.9–5.8) and follow-up of 42.2 months (IQR 23–60). Baseline median eGFR was 58 mls/min; 55% (n = 43) of patients had baseline CKD stage 3 and the remainder stage 1–2. By last follow-up, 1/35 (2.8%) of baseline CKD 1–2, 7/27 (25.9%) CKD 3a and 11/16 (68.8%) CKD 3b had developed CKD stage 4–5. The estimated probability of freedom from CKD stage 4–5 at 1 and 5 years was 89.6% (CI 83.0–97.6) and 65% (CI 51.4–81.7) respectively. On univariable analysis, worse baseline CKD (*p* < 0.0001) and multi-fraction SABR (*p* = 0.005) were predictive for development of stage 4–5 CKD though only the former remained significant in multivariable model.

**Conclusion:**

In this elderly cohort with pre-existing renal dysfunction, SABR achieved satisfactory nephron sparing with acceptable rates of severe to end-stage CKD. It can be an attractive option in patients who are medically inoperable.

## Introduction

Stereotactic ablative body radiotherapy (SABR) has emerged as a non-invasive treatment modality in the management of primary RCC [1]. It is a potential treatment option for primary localised RCC in patients unsuitable for radical (RN) or partial nephrectomy (PN) [2], particularly those with baseline CKD and comorbidities where RN puts them at high risk of progression to severe CKD. Excellent local control rates following SABR have been demonstrated by multiple authors [3]. Recently, Siva et al. reported a 5-year cumulative local relapse rate of 5.5% in 190 patients treated with SABR for primary RCC across 12 institutions in Australia, Japan, Europe, Canada and the USA [4], and found a median decline in eGFR of 14.2 ml/min/1.73 m^2^. SABR has also been shown to have minimal impact on patient-reported QOL outcomes in a prospective patient cohort [5].

Patients treated with SABR tend to be older than in surgical series, with a median age above 70 [4]. Increasing age is associated with a higher burden of comorbidities, including hypertension, diabetes mellitus and mild-moderate CKD, all independent risk factors for progressive CKD. Though the post-SABR decline in renal function is minimal and generally acceptable for most patients with primary RCC, this decline can be significant in certain patients with multiple risk factors.

Increasing age and type of surgery (radical vs partial nephrectomy), tumor size, hypertension and diabetes mellites have been consistently shown to be independent predictors of progressive CKD following surgical management of primary RCC [6–10]. In a Canadian retrospective study, the incidence of end-stage renal disease (ESRD) in patients with baseline CKD stage 3 was 9.4% (13/139) and 2.2% (2/93) after RN and PN, respectively [9]. We hypothesise that the presence of baseline renal insufficiency, solitary kidney, increasing age, increasing tumour size, diabetes and hypertension can also result in reduced tolerance of the kidney to radiotherapy. However, the contribution of these factors is unclear as there has been no previous published data predicting the progression of CKD following SABR for primary RCC. This study aims to assess the incidence of severe-end-stage CKD (stage 4–5) and the clinical factors predicting its development in patients treated with SABR for primary RCC.

## Methods

This project was approved by the Peter MacCallum Cancer Centre (PMCC) ethics review committee (QA/77039/PMCC). Adult patients (above 18 years old, no upper age limit) with biopsy-proven or radiologically diagnosed primary renal cell carcinoma (RCC) receiving SABR at the PMCC radiotherapy department between 2012 and 2020 for whom consensus treatment recommendations had been made in our weekly multidisciplinary meetings were included. Only patients with no systemic metastatic disease, baseline eGFR of ≥ 30 ml/min/1.73 m^2^, and at least one post-SABR eGFR at six months or later were included in this analysis. Patients with upper tract urothelial carcinoma were excluded. All patients had baseline blood investigations and computed tomography (CT). SABR is mostly recommended in medically inoperable patients or ones at high risk to progress to ESRD post-surgery.

A radiotherapy dose of 26 Gy in a single fraction for tumours smaller than 4 cm and 42 Gy in 3 fractions for those ≥ 4 cm was delivered on a linear accelerator (Varian Medical Systems, Palo Alto, Ca). All patients underwent a four-dimensional CT scan (4DCT) in free-breathing with a BodyFix vacuum drape (Elekta, Stockholm Sweden). Internal target volume (ITV) was contoured encompassing motion derived from 4DCT. A planning target volume (PTV) was generated from an isotropic 5 mm margin expansion of ITV. The radiotherapy treatments were planned with 3-D conformal (until early 2016) or intensity modulation techniques (IMRT/VMAT), aiming to cover 99% of the PTV by 100% of the dose (D_99_PTV = 100%). Lower PTV coverage (D_95_PTV = 95%) was accepted where necessary to meet OAR dose constraints. A peak dose between 125–143% was allowed. The OAR dose constraints are based on FASTRACK protocol [11]. In patients receiving three fractions, treatment was delivered on non-consecutive days [11]. All patients underwent daily online image verification with a Cone Beam CT (CBCT) with matched to soft tissue, GTV/PTV.

After completion of SABR, patients were reviewed at four weeks to assess for any treatment-related acute toxicity. Patients were followed-up for ongoing response assessment and treatment-related toxicity every 4–6 months for the first two years, six monthly for years 3–5 and yearly thereafter. Renal function with serum creatinine/eGFR and CT scan were performed each visit.

The study outcome was the development of severe to end-stage CKD (stage 4–5), defined as GFR of < 30 ml/min/1.73 m^2^. The effect of clinical factors, including age, hypertension, diabetes, Solitary kidney, pre-SABR CKD stage, size of the primary tumour and radiotherapy fractions, were assessed. “Post SABR eGFR” was defined as the final eGFR available for each patient. The Chronic Kidney Disease-Epidemiology Collaboration (CKD-EPI) equation was used to calculate eGFR.

### Statistical analysis

All statistical analyses were performed using R Statistical Software (v4.2.1). Patient and tumor characteristics are summarized: categorical variables were reported as frequency and percentage, continuous variables were presented as either medians with an interquartile range (IQR) or means and standard deviation (SD). Non-parametric statistical tests—Fisher’s exact test for categorical variables and Kruskal–Wallis test for continuous variables—were used.

Kaplan–Meier curves with log-rank tests was used to estimate the probability of freedom from the development of CKD stage 4–5 from the date of SABR. Censoring was done at the date of last follow up. Univariable and multivariable Cox proportional hazard models were constructed to identify predictive factors for the development of CKD stage 4–5. Hazard ratios with 95% confidence intervals (CI95) and *p* values were calculated. Statistical significance is defined at *p* < 0.05.

## Results

A total of 78 patients who met the inclusion criteria were enrolled in this study. Table 1 provides a summary of the baseline characteristics of the patients. The median age at the time of SABR was 77.8 years (IQR: 70.4–82.6). Of the patients, 73% (n = 57) were male, and 54% (n = 42) had right-sided kidney tumors. Biopsy confirmation was obtained in 67 (85.9%) patients with 63 (94%) demonstrating clear cell histology. Most of the patients (64%, n = 50), had T1b tumors, and the median tumor size was 4.5 cm (IQR 3.9–5.8). Hypertension and diabetes were present in 79% (n = 62) and 49% (n = 38) of the patients, respectively. Pre-treatment split renal function assessments were available in 64 (88.9%) out of 72 patients with dual kidneys. The median split function was 49.5%: 50.5% for the target to the contralateral kidney.

**Table 1:** Patient characteristics

Patient characteristic	All patients (n = 78)
Age at SABR (years)	
Median (IQR)	77.8 (70.4–82.6)
Gender	
Male	57 (73%)
Female	21 (27%)
ECOG PS	
0	36 (46%)
1	25 (32%)
2	17 (22%)
Pathological confirmation	67 (85.9%)
Tumor size (cm)	
Mean (SD)	4.8 (1.3)
< 4	25 (32%)
≥ 4	53 (68%)
Tumor side	
Left	36 (46%)
Right	42 (54%)
Solitary versus dual kidneys	
Dual kidneys	72 (92.4%)
Single kidney	6 (7.6%)
Ipsilateral (Target) kidney percentage function median (IQR)	49.5% (46–52%)
Baseline CKD stage	
1–2	35 (44.9%)
3a	27 (34.6%)
3b	16 (20.5%)
T stage	
1	70 (90%)
2	5 (6%)
3	3 (4%)
Hypertension	
Yes	62 (79%)
No	16 (21%)
Diabetes mellites	
Yes	38 (49%)
No	40 (51%)
SABR fractions	
Single	31 (40%)
Multiple	47 (60%)

As only 3 patients had CKD stage 1, they were combined with CKD stage 2 for the purpose of analysis. Among all patients, the median eGFR was 58 (IQR: 45.3–69.8), while the median eGFR for CKD stage 1–2, 3a, and 3b was 70 (IQR: 64.5–85.5), 51 (IQR: 47.5–55.5), and 37.5 (IQR: 33.5–41.2) ml/min/1.73 m^2^, respectively (Table 2). Thirty-one patients (40%) received single-fraction SABR, with a higher proportion of CKD stage 1–2 patients receiving this treatment (n = 21, 60%) compared to CKD stage 3b patients (n = 3, 19%, *p* = 0.005).

**Table 2:** Mean and median pre and post SABR GFR, stratified by initial CKD stage

	Pre-SABR eGFR		Post-SABR eGFR	
	Mean (SD)	Median (IQR)	mean	Median
*Pre-SABR CKD stage*				
Stage 1–2	74.1 (10.8)	70 (64.5–85.5)	57.7 (19.1)	55 (41.5–74)
Stage 3a	51.3 (4.8)	51 (47.5–55.5)	39.5 (11.9)	39 (30–50)
Stage 3b	37 (4.5)	37.5 (33.5–41.2)	22.6 (8.8)	23.5 (14.8–31)

**Fig. 1:**
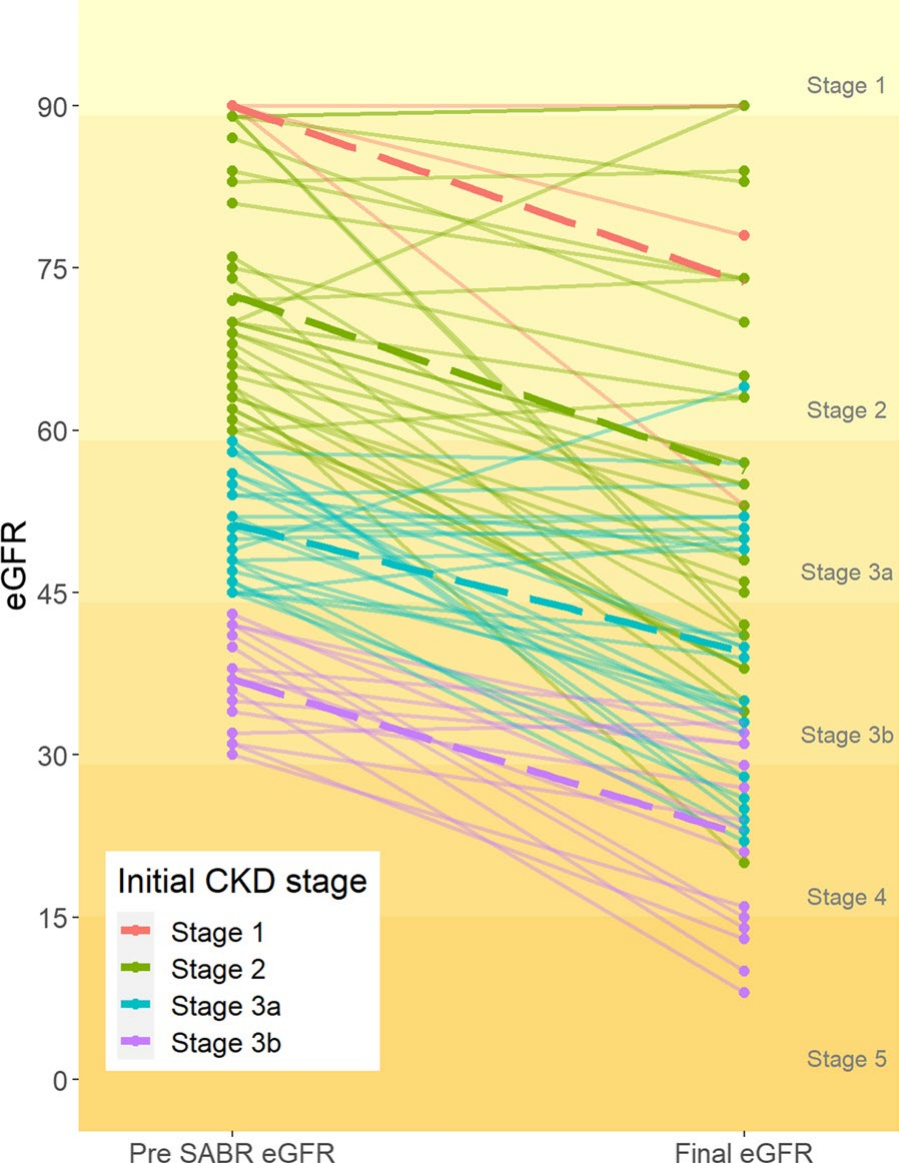
Profile plot demonstrating the trajectory of renal function pre- and post-SABR. Each line represents one patient, with line colours denoting their pre-treatment CKD stage. Dotted lines illustrate fitted linear models, stratified by initial CKD stage

For all patients, median eGFR decline from baseline was 14 (IQR 4.25–23.50) ml/min/1.73 m^2^. The last available median eGFR post-SABR for initial CKD stage 1–2, 3a, and 3b was 55 (IQR: 41.5–74), 39 (IQR: 30–50) and 23.5 (IQR: 14.8–31) ml/min/1.73m^2^, respectively (Table 2). The trajectory of renal function decline following SABR is visualised in Fig. 1.

**Fig. 2:**
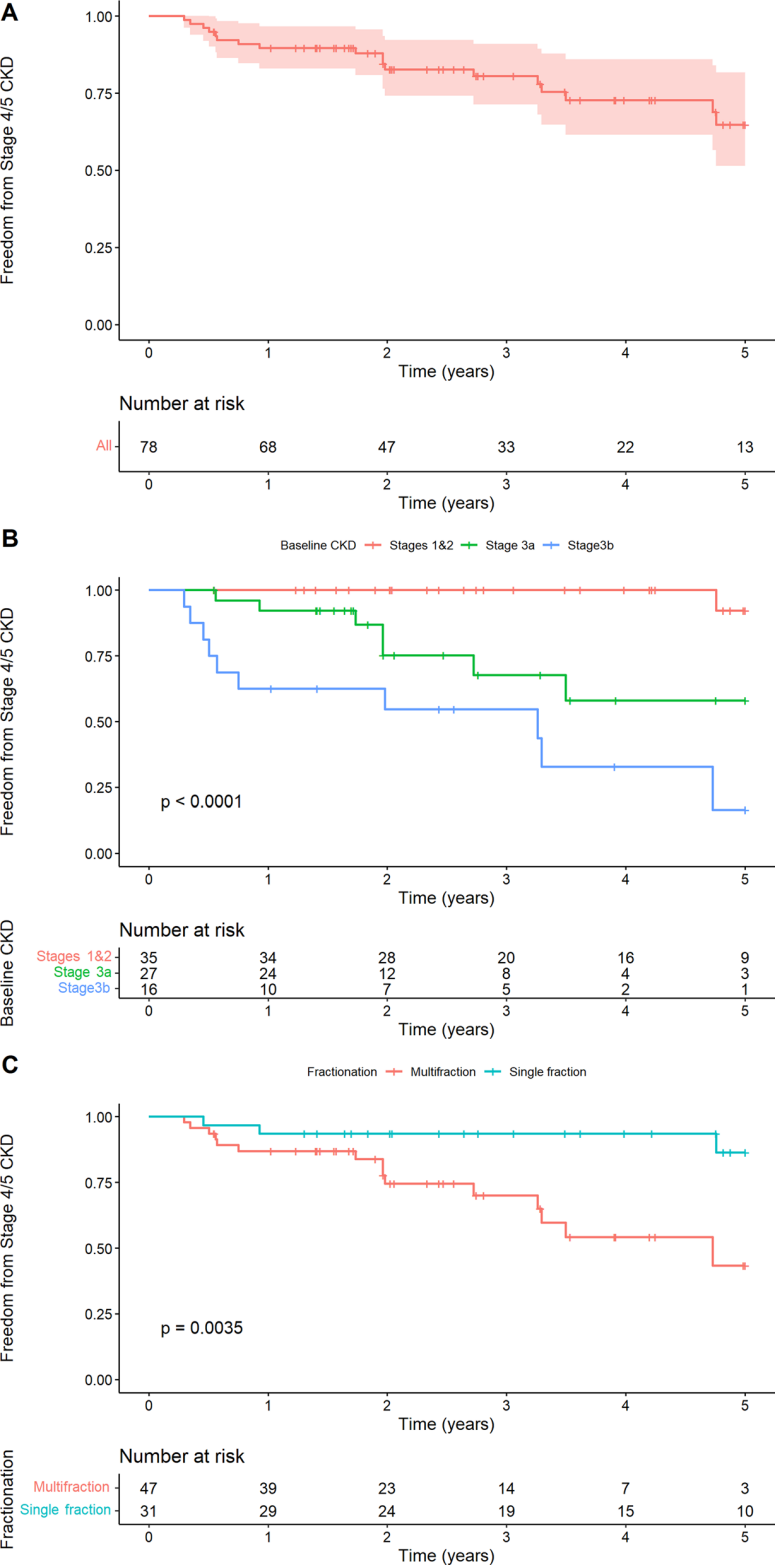
Probability of freedom from CKD stage 4–5 across **A** all patients, **B** stratified by baseline renal function and **C** by fractionation schedule

By their last follow-up (median 42.2 months), 19 patients (24%) developed CKD stage 4–5, four of whom developed end-stage renal disease (CKD stage 5). The Kaplan–Meier estimated probability of freedom from the development of CKD stage 4–5 at 1 and 5 years was 89.6% (CI 83.0–97.6) and 65% (CI 51.4–81.7), respectively (Fig. 2A).

One of 35 patients with CKD stage 1–2 developed CKD stage 4–5, while 7/27 and 11/16 patients with CKD stage 3a and 3b, respectively, progressed to CKD stage 4–5. The 1- and 5-year probability of freedom from the development of CKD stage 4–5 were 100% and 92.3%, 92.3% and 58.1%, 62.5% and 16.4% for patients with baseline CKD stage 1–2, 3a and 3b, respectively (log-rank *p* < 0.0001, Fig. 2B). Patients who received single-fraction SABR had a lower probability of developing CKD stage 4–5 (*p* = 0.0035 by log-rank test) than those who received multi-fraction SABR (Fig. 2C).

**Fig. 3:**
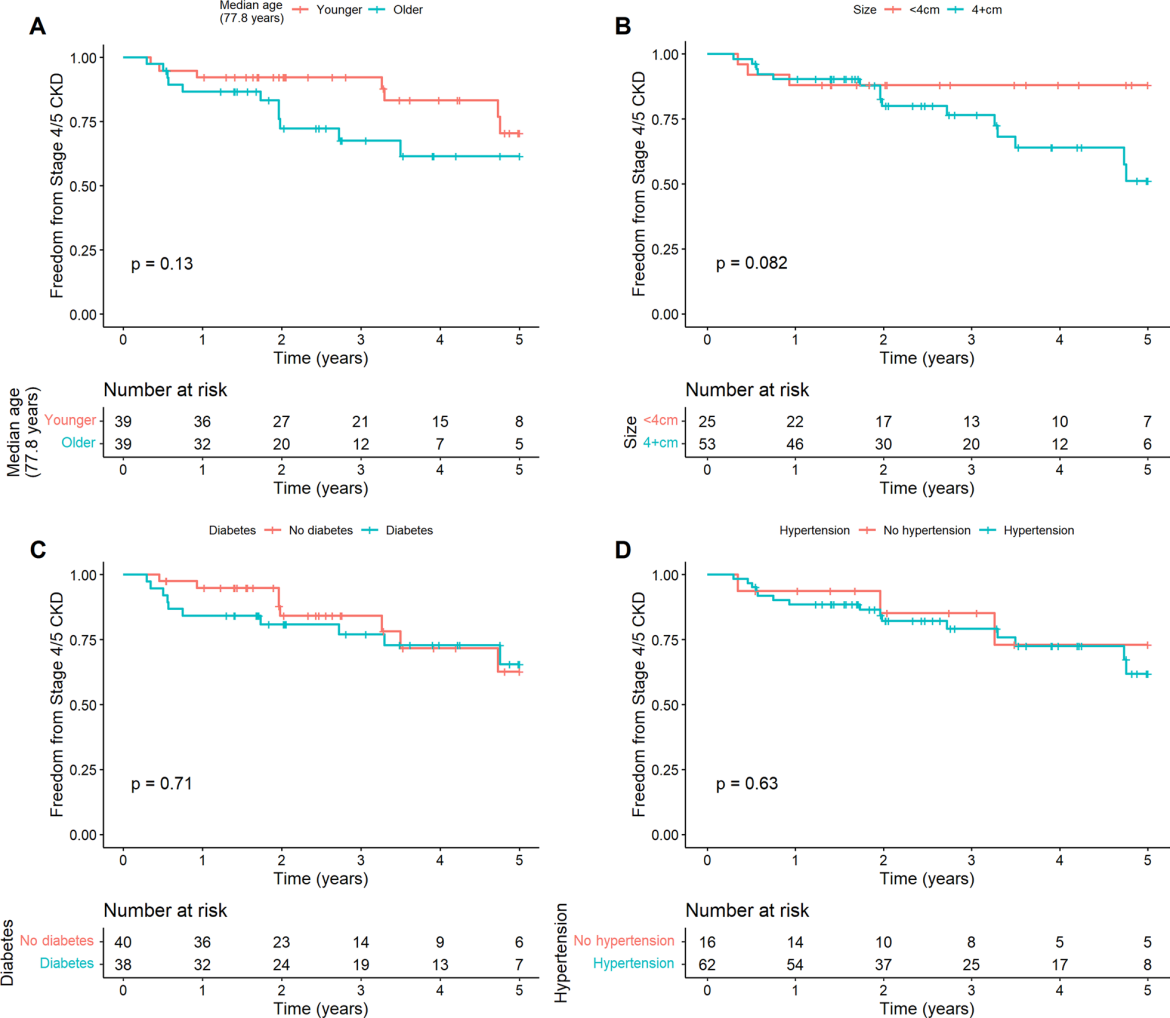
Probability of freedom from CKD stage 4–5 across stratified by **A** median age, **B** size and by the presence of comorbid **C** diabetes and **D** hypertension

Although the numerical difference in the risk of developing CKD stage 4–5 was lower in younger patients than those older than the median age, the difference was not statistically significant (*p* = 0.13 by log-rank test) (Fig. 3A). Neither diabetes mellitus, solitary kidney, hypertension, nor tumor size (< 4 cm vs. ≥ 4 cm) was statistically significant (Figs. 3B–D). In a multivariable Cox proportional hazards model, baseline CKD (stage 1–2 vs. 3a and stage 1–2 vs. 3b) remained statistically significant (Table 3).

**Table 3:** Factors contributing to the development of CKD stage 4–5 in a Cox proportional hazard model, univariable and multivariable analyses

Baseline variable	Univariate		Multivariate	
	Hazard ratio (95% CI)	*P* value	Hazard ratio (95% CI)	*P* value
*CKD group*				
Group 2 versus group 1	13.8 (1.68–113)	0.015	9.7 (1.05–88.8)	0.045
Group 3 versus group 1	43.3 (5.52–340)	0.000	31.2 (3.6–268)	0.002
Single versus multi-fraction SABR	0.22 (0.070–0.69)	0.009	0.51 (0.14–1.8)	0.30
Age (< median vs. ≥ median)	2.3 (0.91–6.0)	0.079	1.2 (0.45–3.2)	0.71
Tumour size (< 4cm vs. 4 + cm)	2.2 (0.73–6.8)	0.16	Not applicable	–
Hypertension	1.4 (0.41–4.9)	0.58	Not applicable	–
Diabetes	1.3 (0.53–3.3)	0.56	Not applicable	–
Dual versus solitary kidney	0.7 (0.1–5.1)	0.7	Not applicable	–

## Discussion

To the best of our knowledge, this is the first study evaluating clinical factors predictive of CKD stage 4–5 post-SABR for primary RCC. It is also one of the largest single institution series of renal SABR reported to date. Our series demonstrates post-treatment deterioration in renal function comparable to published surgical series despite our older cohort (median age of 77.8 years vs. 60–65 in most surgical series) and relatively lower baseline eGFR. Baseline CKD stage remained the only predictive factor on multivariable analysis with 1/35 (2.8%) of patients with baseline CKD stage 1–2 progressed to stage 4 at the last follow-up, with no patients developing ESRD.

The incidence of early-stage renal cell carcinoma (RCC) has increased over the last 3 decades, alongside a concomitant decrease in mortality of RCC as a whole [12]. Considering the excellent long-term oncological outcomes of early stage RCC, recent focus has shifted towards maintaining quality of life (QOL) through the reduction of treatment-related morbidity, in particular renal function preservation. Chronic kidney disease (CKD) is associated with a higher risk of death, cardiovascular events, and hospitalization. In a large population-based study, estimated glomerular filtration rates (eGFR) of 15–29 and < 15 ml/min were associated with age-adjusted mortality rates of 11.4 and 14.1 per 100 person-years, respectively [13]. It is therefore crucial to limit CKD progression following treatment for localized early-stage RCC.

Multiple studies have addressed CKD progression post-surgery in patients with baseline CKD stage 1–2 and reported better outcomes with PN. A large Canadian retrospective series by Mason et al. reported 2.1% (10/466) and 5.4% (29/532) of patients with baseline CKD stage 1–2 progressing to stage 4–5 following PN and RN, respectively [9]. Similar findings were found in another large retrospective study in RCC patients with baseline CKD stage 2 (CKD stage 4–5: 2.7% in PN and 4.3% in RN) [14]. In the only randomized trial of RN versus PN (EORTC 30904), postoperative incidence of CKD stage 4–5 incidence was significantly lower in the PN group (6.3% vs. 10.0%) [15].

In previously reported surgical series, preoperative CKD stage was significantly associated with post-operative CKD progression [9, 16]. Mason et al. reported worse progression with increasing CKD stage for patients treated with both PN and RN—in patients with CKD stage 2 at baseline, eGFR fell below 30 in 8.4% of the RN and 3% of the PN group at last follow-up, but 43.2% (60/139) and 19.4% (18/93) of patients with baseline CKD stage 3 progressed to stage 4–5 following RN and PN, respectively [9]. In our study, baseline CKD stage was also the strongest predictor of progression to stage 4–5 on univariable and multivariable analysis, with stage 3b patients faring the worst—83.9% developed CKD stage 4–5 by their last follow up. The interpretation of this is challenging as the natural history of CKD is highly variable even in the absence of renal cell carcinoma; CKD progression risk does however seem to be higher in stage 3b patients compared to patients with earlier stage disease [17–19]. Our cohort shows that whilst the general trend was for a decline in renal function over time following SABR, 13/78 patients (17%) had a final eGFR higher than the baseline eGFR. However, this was marginal—the increase in eGFR was < 5 in all but 3 patients (pre-SBRT eGFR 49,45 and 70), which might be due to compensatory responses from the remaining functional nephrons.

Patients with baseline CKD stage 3b and primary RCC present a significant dilemma to the treating physician. Any intervention has the potential to significantly accelerate renal function decline in this already high-risk cohort. One study by Takagi et al. reported no significant difference in development of new onset CKD stage 4–5 in patients with base line CKD 3b at 2-years with PN (55%) over RN (46%) [20]. In our study, we also found an ongoing decline of renal function following SABR for patients with baseline CKD 3b, with 1- and 5-year freedom from CKD stage 4–5 development of 58.1% and 16.4%, respectively. Four patients (all stage 3b at baseline) progressed to ESRD at last follow up, consistent with the long-term follow-up results post SABR for RCC previously reported by Siva et al. [4]. In that study, 3.7% (7/190) patients with mean ± SD baseline eGFR of 32.8 ± 13.2 mL/min, underwent dialysis post-SABR. Therefore, early education is paramount in this high-risk RCC cohort to ensure that they are adequately informed to make the appropriate choices, including the option of best supportive care. Irrespective of treatment modality, patients in this group should be offered early nephrology referral for regular monitoring and intervention to slow the progression of renal function decline and management of CKD-related complications.

Whilst increasing age, hypertension, diabetes mellitus and tumor size have been linked with greater loss in renal function and progression to CKD stage 4–5 in surgical series [6–8, 21], these factors were not significant contributors to renal function loss in this study. This might be due to a smaller sample size, older population or poorer baseline renal function at the time of SABR in this study compared to previous surgical series, overwhelming the impact of comorbidity on post-treatment renal function. It may also reflect the different pathogenesis of nephropathy post-surgery and radiotherapy. However, similar findings were reported in prior radiotherapy studies as well. In one study, Park et al. [22] found only baseline eGFR to be the sole prognostic factor for renal function impairment in their multivariable logistic regression analysis when evaluating the risk factors for renal function impairment in patients with gastric cancer treated with surgery and adjuvant radiotherapy.

Renal parenchyma loss remains the main cause of renal function decline post-PN, but renal ischemia secondary to renal artery clamping during surgery is also a contributing factor, with prolonged ischemia time associated with more postoperative renal function decline [23, 24]. Diabetes has been demonstrated to increase reperfusion injury following ischemia in a rodent model [25] and whilst the aetiopathogenesis of renal failure in the setting of hypertension following renal artery clamping is less clear, there are well appreciated complex interactions between hypertension and renal ischemia [26]. By comparison, radiation nephropathy results in cell death through the dose-dependent creation of double-stranded DNA breaks [27]. There should in theory be no effect on nephrons not receiving a significant radiation dose and the severity of radiation nephropathy therefore depends on the total dose and the kidney volume being irradiated [27, 28]. The steep dose gradient in SABR limits the volume of irradiated normal tissue and results in reduced radiation dose to normal renal parenchyma. This is consistent with our observation that there is an almost identical decline in mean eGFR in our cohort, irrespective of baseline CKD stage (Fig. 1).

In this study, the single fraction SABR regimen was associated with a lower incidence of CKD stage 4–5 compared to multi-fraction SABR on univariable analysis (*p* = 0.005), though this did not reach statistical significance on multivariable analysis. Whilst we cannot draw firm conclusions from the data due to sample size and imbalances between fractionation groups, we might hypothesise that the lower total dose used in single fraction SABR results in reduced dose to normal renal parenchyma compared to multi-fraction, better preserving renal function. In one study, Siva et al. [29] reported that limiting the volume of kidney receiving > 50% prescription dose may reduce the risk of renal function loss and this is backed up by Yamamoto et al. [30] who found strong correlations between the dose distribution of 20 and 30 Gy and renal atrophy in patients receiving SABR. The choice of fractionation schedule is however heavily confounded by other factors, including age, tumour size and baseline CKD status. Further investigation of the impact of fractionation schedule on CKD progression is necessary, especially considering previous work showing worse local failure and progression free survival in multi-fraction SABR schedules [4].

Despite being the first study of its kind, there are also a few limitations. (1) This is a retrospective study. (2) Our sample size is relatively small compared to most published surgical series. Both limitations result from the novelty of SABR as a treatment modality for localised primary RCC and limited indications for its use in patients who are medically inoperable or at high risk of dialysis post-surgery. (3) Renal parenchyma dosimetry data is not presently available for these patients.

To address these limitations, we have established an international prospective registry on the platform of International Radiosurgery Oncology Consortium for Kidney cancer (IROCK), which will continue to collect prospective data (https://www.irockregistry.com/). We are also ensuring that dosimetry data is included for this ongoing project. In planning for future randomised trials, the effect of single vs multifraction SABR should be considered, with tumor related outcomes and renal function changes as co-endpoints.

## Conclusion

Progression to severe chronic kidney disease post SABR for primary RCC is comparable to historical other treatment modalities. Pre-operative CKD stage remains the strongest predictor for the probability of progression to severe or end stage CKD with negligible risk in patients with baseline eGFR > 60 ml/min/1.73 m^2^, making SABR an attractive alternative in certain scenarios of medically inoperable elderly patients with co-morbidities. Future randomised studies should assess the significance of SABR fractionation on renal function.

## Data Availability

The datasets generated and/or analysed during the current study are not publicly available due to local laws but are available from the corresponding author on reasonable request.
